# Chimaerin Suppresses Rac1 Activation at the Apical Membrane to Maintain the Cyst Structure

**DOI:** 10.1371/journal.pone.0052258

**Published:** 2012-12-20

**Authors:** Shunsuke Yagi, Michiyuki Matsuda, Etsuko Kiyokawa

**Affiliations:** 1 Laboratory of Bioimaging and Cell Signaling, Graduate School of Biostudies, Kyoto University, Yoshida Konoe-cho, Sakyo-ku, Kyoto, Japan; 2 Department of Pathology and Biology of Diseases, Graduate School of Medicine, Kyoto University, Yoshida Konoe-cho, Sakyo-ku, Kyoto, Japan; 3 Department of Oncologic Pathology, Kanazawa Medical University, Uchinada, Kahoku-gun, Ishikawa, Japan; Astar-Neuroscience Research Partnership (NRP) and Institute of Medical Biology (IMB), Singapore

## Abstract

Epithelial organs are made of a well-polarized monolayer of epithelial cells, and their morphology is maintained strictly for their proper functions. Previously, we showed that Rac1 activation is suppressed at the apical membrane in the mature organoid, and that such spatially biased Rac1 activity is required for the polarity maintenance. Here we identify Chimaerin, a GTPase activating protein for Rac1, as a suppressor of Rac1 activity at the apical membrane. Depletion of Chimaerin causes over-activation of Rac1 at the apical membrane in the presence of hepatocyte growth factor (HGF), followed by luminal cell accumulation. Importantly, Chimaerin depletion did not inhibit extension formation at the basal membrane. These observations suggest that Chimaerin functions as the apical-specific Rac1 GAP to maintain epithelial morphology.

## Introduction

Epithelial organs, such as kidney, mammary gland, salivary gland, and pancreas, contain cysts and tubules, which are both lumen-enclosing structures, although tubules are cylindrical instead of spherical [1]. Observing these structures in the living animal is technically challenging. Recently, three-dimensional cell culture in gels of extracellular matrix (ECM) allowed a close approximation of the biological situation *in vitro*. Madin Darby canine kidney (MDCK) cells form cysts consisting of a monolayer of polarized cells surrounding a lumen in Matrigel gels; therefore, they have been used to investigate molecular mechanisms for polarity and morphology [1].

By applying Raichu Förster Resonance Energy Transfer (FRET) biosensors to the MDCK cyst system, we recently visualized Rac1 activation during cystogenesis [2]. Rac1 activation was uniform in the entire plasma membrane of MDCK cysts in immature cysts, whereas in mature cysts, the activity became lower at the apical membrane than the lateral membrane. We also set up the system to induce activation or inactivation of the small GTPase Rac1 in different stages of polarity maturation, and found that the forced activation of Rac1 at the apical membrane disrupted apico-basal polarity to reorient cell division axes into the luminal space. In these cysts, cells accumulated in the lumen, which resembles hyperplasia *in vivo*. In contrast, suppression of Rac1 at the basolateral plasma membrane did not significantly affect the morphology of mature cysts. Therefore, it is now established that Rac1 suppression at the apical membrane is required for the mature cyst to maintain its morphology. What remained to be solved is which molecules suppress Rac1 activity at the apical membrane.

Rac1 belongs to the Rho family of small GTPases, which work as intracellular molecular switches and activate their downstream signaling molecules upon perception of external or internal cues [3]. GTP-bound, but not GDP-bound, small GTPases are able to bind to and activate effector proteins. Cycling between GDP- and GTP-bound states is controlled primarily by two classes of regulatory molecules: GTPase-activating proteins (GAPs), which enhance the relatively slow intrinsic GTPase activity of small GTPases; and guanine nucleotide-exchange factors (GEFs), which catalyze the exchange of GDP for GTP *in vivo*. Various GEFs and GAPs have been identified for Rho family GTPases, and their targets overlap [4]; [5], [6]. Since GAPs for Rho family GTPases share a conserved domain, it is not possible at this moment to predict their substrates by their amino-acid sequences [7]. Moreover, discrepancies have been reported between *in vitro* and *in vivo* specificities [6]. Therefore, to identify the GEF responsible for morphology, RNA interference (RNAi) screening was utilized [8], [9]. This method, however, requires enormous work to cover all of the GAPs for Rac1.

In this work, comparing messenger RNA levels between early and late stages of cystogenesis, we first identified which GAPs for Rho family GTPases become expressed in the late stages. Focusing on the lipid distribution assessed by FRET biosensors, we narrowed the list of candidates down to Chimaerin as a suppressor of Rac1 activity at the apical membrane. Chimaerin localized to the apical membrane, probably by diacylglycerol binding, and depletion of Chimaerin caused Rac1 activation there, followed by luminal cell accumulation in the presence of hepatocyte growth factor. These data indicate that Chimaerin is the specific GAP that suppresses Rac1 activation at the apical membrane to maintain the cyst structure.

## Methods

### Plasmids

Plasmids for FRET biosensors Raichu-Rac1 have been reported previously [2]. CFP of from the original FRET biosensor for DIGDA and Pippi [10]–[12] was replaced with TFP, and cloned into the pCX4neo retroviral vector. Mouse β2-chimaerin cDNA was amplified from pEF-BOS-HA-2-chimaerin (a gift from Dr. Hironori Katoh, Kyoto University), and cloned into a pCX4bsr-EGFP plasmid. For shRNA-mediated knockdown experiments, shRNA sequences were: gattatgtccggttatgta (shLuc), ggtgtgaatactgtgctaact (shChn1), and gctggaagagtgattaataaa (shChn2). Corresponding DNA oligomers were cloned into pSUPER vector (Oligoengine, Seattle, WA).

### Cyst Culture

MDCK cells were purchased from RIKEN BioResource Center (No. RCB0995), and maintained in minimal essential medium (MEM) containing Earle’s balanced salt solution (GIBCO) supplemented with 10% fetal bovine serum (Equitech-Bio), 3% L-Gln, 0.1% non-essential amino acids, 1 mM sodium pyruvate, 100 units/ml penicillin, and 100 µg/ml streptomycin, in a 5% CO_2_ humidified incubator at 37°C.

### Retroviral Gene Transfer

To establish the MDCK cells stably expressing FRET biosensors, GFP-β2-chimaerin, dKeima, and shRNAs, in their respective retroviral expression vectors were packaged in BOSC23 cells co-transfected with the packaging plasmid pGP and the envelope plasmid pCMV-VSV-G-Rsv-Rev (provided by Hiroyuki Miyoshi and Atsushi Miyawaki, RIKEN). After infection of MDCK cells with the respective viral stocks, the cells were subjected to selection for 2 days with 2 mg/ml of G418 for pCX4neo vectors, 2 µg/ml of puromycin for pCX4puro vectors, and 10 µg/ml of blasticidin for pCX4bsr vectors.

### HGF Stimulation

Conditioned medium containing HGF produced by MRC-5 cells has described previously [2]. In this study, 1X conditioned medium was used for HGF stimulation.

### Microarray Analysis

To prepare cysts in early and late stages, MDCK cells were cultured with Matrigel for 2 and 10 days. Cysts in Matrigel were incubated with 1.25 mM EDTA/phosphate buffered saline (PBS) on ice for 1 hour to depolymerize the Matrigel, followed by washing three times with PBS. The cysts were centrifuged and stored in liquid nitrogen. RNA purification and microarray experiments [Canine (V2) Gene Expression Microarray (Agilent Technologies)] and analysis [Agilent Feature Extraction] were performed by Takara Bio Inc (Shiga, Japan).

### Quantitative PCR

Total RNA was purified with an RNeasy Micro Kit (QIAGEN, Hilden, Germany) and was reverse-transcribed using a High Capacity cDNA Reverse Transcription kit (Applied Biosystems, Foster City, CA) according to the manufacturer’s protocol. mRNA Expression of Chn1, Chn2, and GAPDH mRNAs was analyzed by Power SYBR Green PCR Master Mix (Applied Biosystems) with an ABI PRISM7300 Sequence Detection System (Applied Biosystems). Primers used for quantitative PCR were: Chn1 forward, gccgcgttgaacgatatac; Chn1 reverse, tcttcgtttttgataagcagctc; Chn2 forward, gccactctactccgagaaaaag; Chn2 reverse, cgcttgtttttcttgattcattt; GAPDH forward, tccctcaagattgtcagcaa; and GAPDH reverse, tggatgactttggctagagga.

### Confocal Microscopy

Details of imaging and image acquisition settings were described in a previous report [2]. Briefly, cysts on Matrigel were placed on glass-bottom dishes containing CO_2_-independent medium (GIBCO) containing 2% Matrigel, and imaged with an IX81 inverted microscope (Olympus) or BX upright microscope equipped with an FV1000 confocal imaging system (Olympus).

**Figure 1:**
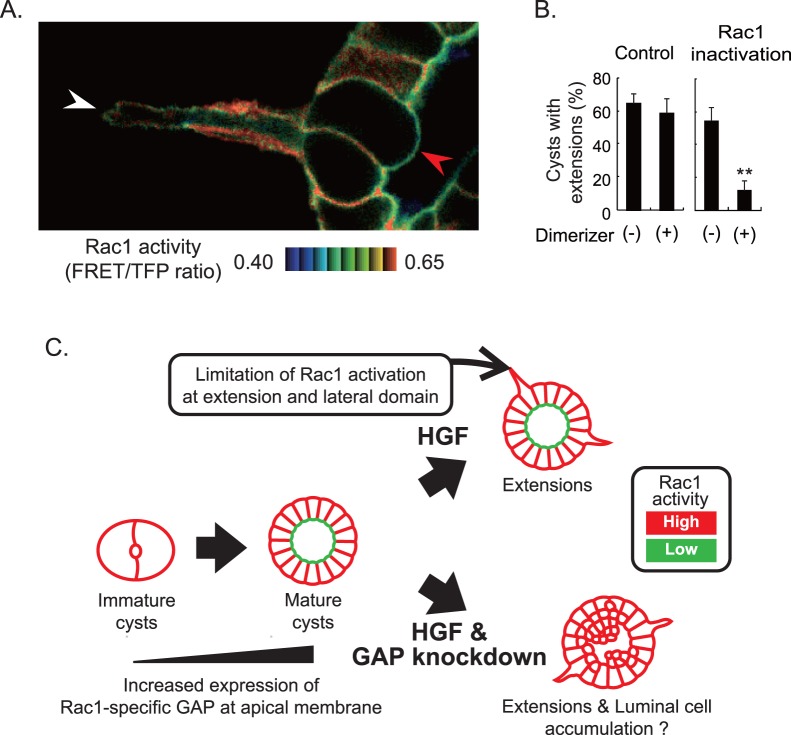
Spatio-temporal regulation of Rac1 activity. (A) MDCK cells expressing Raichu-Rac1 were cultured to form cysts for 10 days, treated with HGF for 12 hours, and imaged with an upright confocal microscope. White and red arrowheads indicate an extension and the apical membrane, respectively. The calculated FRET efficiency was colored in intensity-modulated display (IMD) modes as shown at the bottom. The upper and lower limits of the ratio range are shown at the bottom. (B) MDCK cysts expressing Lyn-FRB alone (Control) or with FKBP fused to the GAP domain of β2-chimaerin (Rac1 inactivation) were treated with HGF and Dimerizer for 12 hours (Supplemental Fig. S1). Shown are the averages of three independent experiments with standard deviation (SD). The numbers of scored cysts are: Experiment (Exp.) 1 [n = 53 (control, Dimerizer (−)), 76 (control, Dimerizer (+)), 100 (Rac1 inactivation, Dimerizer (−)), 88 (Rac1 inactivation, Dimerizer (+))]; Exp. 2 [n = 104, 106, 79, 70]; Exp. 3 [67, 97, 101, 84]. **P<0.01. (C) Schematic view of the concept of this study (see the main text).

For imaging HGF-induced structures (Figure 1A), samples were examined on a BX upright microscope (Olympus) equipped with an FV1000 confocal imaging system (Olympus) and XLUMPLFL 20X W/0.98. The excitation laser and fluorescence filter settings were as follows: excitation laser for CFP/FRET, 440 nm; excitation dichroic mirror, DM405–440/515; CFP channel PMT dichroic mirror, SDM 510; CFP channel PMT filter, BA465–495; FRET channel PMT dichroic mirror; FRET channel PMT filter, BA520–550 nm.

**Figure 2:**
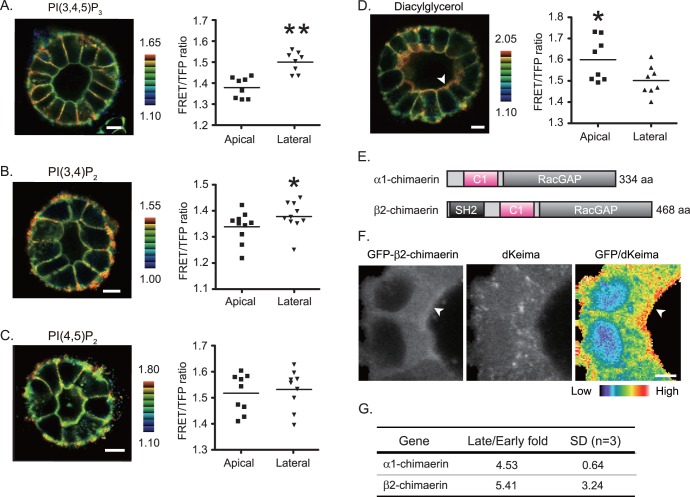
Localization of lipids and Chn2 in cysts. FRET images and quantitative analysis of PI(3,4,5)P_3_ (A), PI(3,4)P_2_ (B), PI(4,5)P_2_ (C) and diacylglycerol (D). The calculated FRET efficiency was colored and shown as in Fig. 1A.The white arrowhead in (D) indicates accumulation of diacylglycerol at the apical membrane. * P<0.05 and ** P<0.01 (E) Schematic view of α1- and β2-Chimaerin encoding 334 and 468 amino acids (AA). SH2 denotes the Src Homology 2 domain. (F) MDCK cysts expressing both dKeima and GFP-β2-Chimaerin were processed and imaged as described in Methods. Localization of GFP-β2-Chimaerin shown in GFP channel (left), dKeima channel (middle) and relative localization of GFP-β2-chimaerin over dKeima (right) shown in pseudocolor mode (at the bottom). White arrowheads indicate apical enrichment of GFP-β2-chimaerin. Scale Bar = 5 µm. (G) Results of quantitative PCR from cysts in early (2 days) and late (10 days) stages. Shown are the averages from three independent experiments with SD.

**Figure 3:**
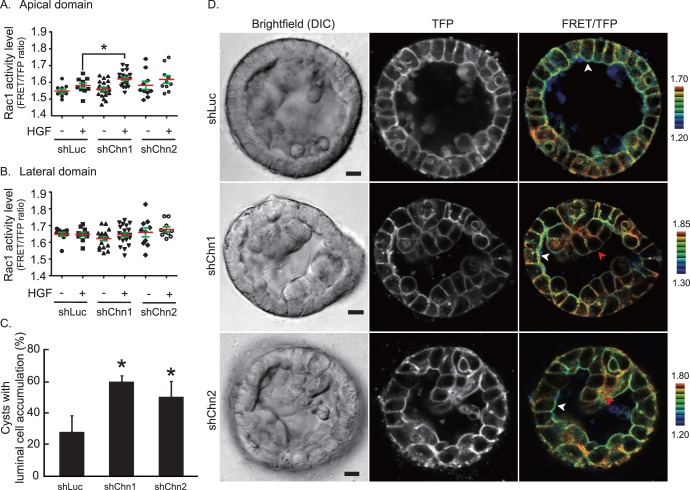
Chn1 and Chn2 knockdown affect Rac1 activity and cyst morphology. MDCK cyst expressing shLuc, shChn1, and shChn2 together with Raichu-Rac1 were stimulated with HGF for 30 minutes. And then Rac1 activity of the cells in the cyst wall was calculated at the apical membrane (A) and the lateral membrane (B). Red and green lines indicate the average of FRET/TFP ratios and SD, respectively. *P<0.05. (C) MDCK cysts expressing shLuc, shChn1, and shChn2 were treated with HGF for 2 days and imaged. Shown are the averages of three independent experiments with SD. The numbers of scored cysts are: Experiment (Exp.) 1 [n = 75 (shLuc), 62 (shChn1), 72 (shChn2)]; Exp2 [n = 80 (shLuc), 55 (shChn1), 66 (shChn2)]; Exp3 [60 (shLuc), 70 (shChn1), 61 (shChn2)]. *P<0.05. (D) Representative images of MDCK cysts expressing shLuc (upper), shChn1 (middle), and shChn2 (bottom) together with Raichu-Rac1. Bright-field images (left column), TFP images (middle column) were obtained with an inverted confocal microscope, and FRET/TFP ratio images (right column) were calculated. Bars indicate 10 µm. White arrowheads indicate low Rac1 activity at the apical domain. Red arrowheads indicate luminal cell accumulation showing high Rac1 activity through the whole plasma membrane.

For imaging cysts (Figure 2A, B, C, D, and Figure 3D), an IX81 inverted microscope (Olympus) equipped with an FV1000 confocal imaging system (Olympus), UPlanSApo 60X O/1.35 was used. The excitation laser and fluorescence filter settings were as follows: excitation laser, 440 nm; excitation dichroic mirror, DM405–440/515; CFP channel PMT dichroic mirror, SDM 510; CFP channel PMT spectral setting, 460–500 nm; FRET channel PMT dichroic mirror; FRET channel PMT spectral setting, 515–615 nm.

### Image Analysis

All fluorescence images and FRET images were processed with MetaMorph software (Universal Imaging, West Chester, PA) and calculated with Excel software (Microsoft) as described previously [2].

### Image Processing for Chn2 Localization

Before imaging, cysts were treated with 0.01% of saponin for 1 minute and fixed with 0.25% PFA/PBS for 30 minutes. After background subtraction from the fluorescence intensity of GFP and Keima, a GFP (500 multiplication)/Keima ratio image was processed and expressed using pseudocolor mode (MetaMorph).

### Statistical Analysis

All statistical analysis used Student’s paired t-test in GraphPad prism software (La Jolla, CA).

## Results and Discussion

### Rac1 Activation Upon Hepatocyte Growth Factor Stimulation

The aim of this study is to understand the role and the mechanism underlying the polarized Rac1 activity in epithelial cells. For this purpose, we employed MDCK cells expressing Raichu-Rac1, a FRET biosensor of Rac1. In Matrigel, MDCK cells grow to form cysts, wherein Rac1 activity is suppressed at the apical plasma membrane. After the maturation of the cysts, MDCK cells were stimulated with Hepatocyte Growth Factor (HGF), which drives MDCK cells initially to extend the basal membrane outward and later to form tubular structures [13]. In HGF-stimulated MDCK cells, Rac1 activity was high at the baso-lateral plasma membrane, including the extended basal membrane, and low at the apical plasma membrane (Fig. 1A, white arrow head). To examine the role of Rac1 in the HGF-induced tubular extension, we utilized a rapamycin-mediated Rac1 inhibition system (Fig. S1) [2]. In this system, rapamycin, called dimerizer hereafter, is used to induce hetero-dimer formation between fragments of mammalian target of rapamycin (FRB) and FK506-binding protein (FKBP). In MDCK cells expressing both FRB tagged with myristylation signal of Lyn (Lyn-FRB) and FKBP fused with the GAP domain of β2-chimaerin (FKBP-GAP), the dimerizer translocates GAP to the plasma membrane and thereby inactivates Rac1. The MDCK cells were first cultured for 12 hours to form cysts and then stimulated with HGF in the presence or absence of the dimerizer. In the control cyst, which expressed only Lyn-FRB, approximately 60% of the cysts showed extensions irrespective of the presence of the dimerizer (Figure 1B, left graph). In the MDCK cells expressing both Lyn-FRB and FKBP-GAP, 55% of the cysts exhibited tubular extension in the absence of the dimerizer, but only 12% did so in the presence of dimerizer (Figure 1B, right graph). This result indicated that Rac1 is required for the HGF-mediated extension formation.

Importantly, Rac1 activity at the apical membrane remained low in the HGF-stimulated MDCK cells (Fig. 1A, red arrow head). Previously, we showed that forced Rac1 activation at the apical membrane causes luminal cell filling [2]. In contrast to this finding, HGF did not induce luminal cell filling. To explain this difference, we speculated that the presence of a Rac1 GAP at the plasma membrane enforces low Rac1 activity even in the presence of HGF (Fig. 1B). The Rac1 GAP would satisfy two criteria: first, the expression increases during cyst maturation; second, the Rac1 GAP is localized at the apical plasma membrane of the cells.

### Screening for Molecules to Regulate Rac1 Activity during Cystogenesis

We searched for the Rac1-specific GAP that fulfilled the aforementioned expression criterion by mRNA microarray analysis. MDCK cells were cultured in the Matrigel for 2 or 10 days before extraction of mRNAs, which were subjected to mRNA microarray analysis. In mammalian cells, there are about 30 proteins that are predicted to possess GAP activity against Rac1 [6]. Thirty-three GAPs for Rho-family GTPases were present in the microarray analysis used in this study (Supplemental Table S1). Seven genes were enriched more than 2-fold in cells isolated from late stages (Table 1).

**Table 1:** GAPs showing changes in mRNA levels between the early and late stages.

Gene Name	Also known as	Log2	Specificity *	Lipid binding domain **
ARHGAP24	FilGAP	2.91	Rac1, Cdc42 [32]	PH
CHN1		2.17	Rac1 [33]	C1 domain [19]
CHN2		1.36	Rac1 [34]	C1 domain [22]
ARHGAP5	P190-B RhoGAP	1.28	RhoA, Rac1, [35]	FF, P-loop NTPase
SRGAP2		1.10	ND	FBAR, SH3 [36]
ARHGAP29	PARG1	1.02	RhoA	ZPH [37]
SLIT-ROBO	SRGAP3	−1.13	Rac, Cdc42 [38]	FBAR, SH3
ARHGAP28		−1.34	ND	Not reported

ND, Not Determined.

* Modified from Table 1 in [6].

** Based on the NCBI database or literature.

### Enrichment of Diacylglycerol at the Apical Membrane and Identification of Chimaerins as Diacylglycerol-binding GAPs

Localization of GAPs is often regulated by inositol phosphates or diacylglycerol (DAG) [6]. To filter the candidate GAPs further by the second criterion, i.e. apical localization, we examined whether or not each GAP binds to these lipids. Before this, we needed to determine which phospholipids or DAG were enriched at the plasma membrane. We previously developed FRET biosensors for these lipids (Figure S1) [11]; [14]; [15]. To use retroviral-mediated gene transfer, the cyan emitting fluorescent protein in these biosensors was replaced by teal fluorescence protein as previously described [2]. We found that phosphatidylinositol (3,4,5) trisphosphate (PIP_3_) and phosphatidylinositol (3,4) bisphosphate [PI(3,4)P_2_] were enriched at the lateral membrane. These observations are consistent with ours [16], and others with a GFP-tagged PH domain [17]. Although the report with a GFP-tagged PH domain showed that PI(4,5)P_2_ is enriched at the apical plasma membrane [17], PI(4,5)P_2_ was found evenly distributed at the entire plasma membrane with the FRET-based biosensor (Fig. 2C). In striking contrast to the phospholipids, DAG preferentially accumulated at the apical membrane (Fig. 2D). Quantitative analysis confirmed the significant bias in the localization of PI(3,4)P_2_, PIP_3_, and DAG between the apical and lateral plasma membranes. Among them, only DAG was enriched at the apical plasma membrane (Figure 2A, B, C, D, right graphs). These observations prompted us to search for GAPs with a DAG binding domain such as a cysteine-rich motif, C1 domain. Among six candidate GAPs in Table 1, a C1 domain was found only in N-Chimaerin and β-Chimaerin [18].

### β2-Chimaerin Localization at the Apical Membrane

The Chimaerin family is composed of two isoforms, α- and β-chimaerins. Each Chimaerin has two alternative splice variants, named α1 and α2-chimaerins, and β1 and β2-chimaerins, respectively. α2- and β2-chimaerins differ from α1 and β1-chimaerins in that the former contain an extended N-terminal region encompassing an SH2-type domain [19]; [20] (Figure 2E). α-chimaerin is also known as CHIMERIN 1 (Chn1), N-chimaerin, ARHGAP2, or RHOGAP2. β-chimaerin is also known as CHIMERIN 2 (Chn2), ARHGAP3, or RHOGAP3. All Chimaerins have a Rac-GAP domain and C1 domain [21]. In accordance with this structure, β2-chimaerin is recruited to the plasma membrane by the interaction of the C1 domain with DAG [22]. By the microarray analysis with dog cDNAs, we found that α1- and β2 chimaerin were expressed in MDCK cells to a detectable level. α2-chimarerin and β1-chimaerin have not been identified in the cDNA database of *Canis lupus familiaris* deposited in the NCBI database, nor with the microarray chip used in this study. We therefore called canine α1-chimaerin and β2-chimaerin Chn1 and Chn2, respectively, and investigated their roles further in this study.

To verify that Chimaerin is localized at the apical membrane, we established a MDCK cell line expressing GFP-tagged mouse Chn2 together with a cytosolic fluorescent red protein dKeima [23], and cultured the cells in Matrigel to form cysts. GFP-Chn2 localized mostly in the cytoplasm, but also accumulated at the apical membrane (Figure 2F, left panel). This plasma membrane accumulation of fluorescence was not observed for dKeima, which served as a control (middle panel). To confirm the plasma membrane accumulation of GFP-Chn2, a GFP/dKeima image was prepared in pseudocolor (Figure 2F, right panel), showing that GFP-Chn2 preferentially localized at the apical membrane. These results strongly suggest that Chimaerin(s) are recruited to the apical membrane by DAG binding, to inactivate Rac1. Our observation of uneven distribution of DAG during cystogenesis is consistent with the notion that upregulation of Chimaerin in the late stages is important for Rac1 inactivation in the mature cysts. Since there was no commercially-available antibody to detect the endogenous dog Chn1 or Chn2, we confirmed upregulation of Chn1 and Chn2 mRNAs by quantitative PCR (Figure 2G, and Fig. S3).

### Suppression of Rac1 Activation by Chimaerin

To examine whether Chn1 and Chn2 suppress Rac1 activation at the apical membrane, we depleted these proteins using a viral-mediated short hairpin RNA (shRNA) interference system. MDCK cells were infected with retroviruses containing shRNA sequences against Chn1 (shChn1), Chn2 (shChn2), or firefly Luciferase (shLuc) as a negative control [24]. The infected cells were selected with antibiotics. Knockdown efficiency was assessed by RT-PCR (Fig. S2). Knockdown MDCK cysts expressing Raichu-Rac1 were stimulated with HGF for 30 minutes or left unstimulated before acquisition of FRET ratio images. In control cysts, upon HGF treatment, Rac1 was activated weakly at the apical membrane (Figure 3A), but not at the lateral membrane (Figure 3B), suggesting that HGF stimulated GEF at the apical membrane. On the other hand, in shChn1 and shChn2 cysts, HGF treatment caused significant Rac1 activation at the apical membrane (Figure 3A), but not at the lateral membrane (Figure 3B). These data demonstrate that Chn1 and Chn2 suppress Rac1 activation upon HGF stimulation by inactivating Rac1 at the apical membrane.

We next examined the effect of knockdown of Chn1 and Chn2 in HGF-stimulated MDCK cysts. Two days after HGF treatment, cysts with luminal cell accumulation were counted. As shown in Figure 3C, 28% of cysts expressing shLuc RNA showed luminal cell accumulation. Knockdown of Chn1 and Chn2 significantly increased the percentage of abnormal cysts up to 59% and 49%, respectively. Similar results were obtained in cysts simultaneously depleted of both Chn1 and Chn2 (Fig. S4). The activation pattern of Rac1 as well as the morphology was visualized in these cysts (Figure 3D). The control cysts with shLuc expression showed a single lumen lined with monolayered cells (the upper row). A small number of cells were found in the lumen, but the FRET level was very low, indicating that these were dead cells (Figure 3D, the top panel). In the Chn1 knockdown or Chn2 knockdown cysts, a number of cells were accumulated in the cysts and formed a multiple layer of cells (Figure 3D, middle and bottom panels). In these multi-layered cells, Rac1 activity was uniform in the entire plasma membrane (red arrow head). Importantly, even in the Chn1 or Chn2-knockdown cysts, cells that maintained the monolayer structure exhibited lower Rac1 activity at the apical membrane than the lateral plasma membrane (white arrow head).

The results of the Chn1 and Chn2 knockdown experiments strongly suggest that Chn1 and Chn2 suppress Rac1 activity at the apical plasma membrane and that aberrant Rac1 activation at the apical membrane causes cells to lose their apico-basal polarity. Since conditional activation of Rac1 at the apical membrane causes luminal cell accumulation via tight junction breakdown, polarity loss, and misorientation of cell division, we speculate that Rac1 activation at the apical membrane caused by Chimaerin depletion utilizes the same pathways for the abnormal morphology [2]. We previously found that similar luminal cell filling was induced by the expression of constitutive active Ras protein in MDCK cysts [25]. For this, cell-cycle progression and anoikis prevention mediated by PI3 kinase were required. Since living cells were observed in the lumen in Chimaerin depleted cysts, it is possible that Chn1 and Chn2 participate in anoikis regulation.

### No Effect of Chn1 and Chn2 Depletion on HGF-induced Extension

**Figure 4:**
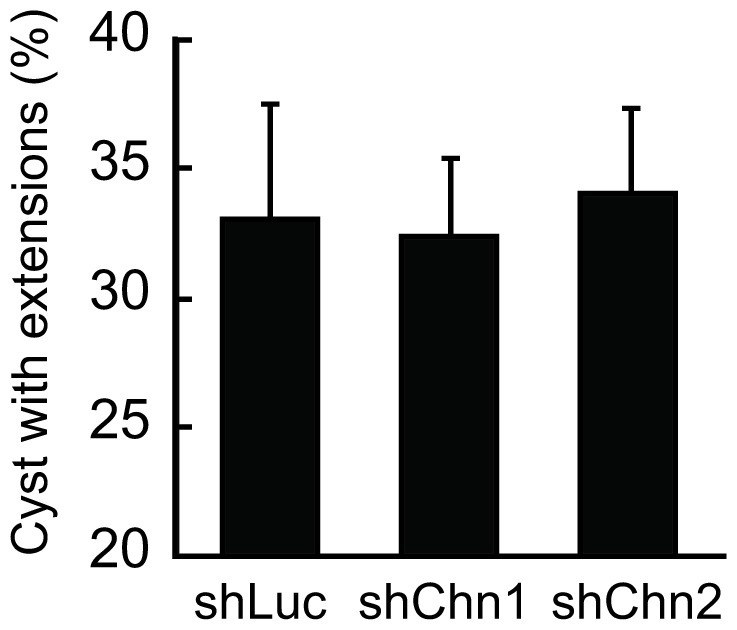
Chn1 and Chn2 knockdown do not affect extension formation. MDCK cysts expressing shLuc, shChn1, and shChn2 were treated with HGF for 12 hours. Cysts with at least one extension were counted. Shown are the averages of three independent experiments with SD. The numbers of scored cysts are: Experiment (Exp.) 1 [n = 34 (shLuc), 36 (shChn1), 38 (shChn2)]; Exp2 [n = 28 (shLuc), 30 (shChn1), 32 (shChn2)]; Exp3 [37 (shLuc), 31 (shChn1), 32 (shChn2)].

Finally, to examine whether Chimaerin functions as the apical-specific GAP, Chimaerin-depleted cysts were treated with HGF, and the numbers of cysts with extensions were counted. As shown in Figure 4, HGF-induced extension was comparable among control (shLuc) and Chimaerin-depleted (Chn1 and Chn2) cysts. These data confirmed that Chimaerin-dependent Rac1 inactivation is limited to the apical membrane.

### Conclusions

Combining microarray analysis data and FRET images of lipid distribution, we found that Rac1-specific GAPs Chn1 and Chn2 are involved in the suppression of Rac1 activity at the apical plasma membrane of the mature cyst (Fig. 1C). GFP-tagged Chn2 was detected at the apical membrane (Fig. 2F), and depletion of Chn1 and Chn2 increased Rac1 activity at the apical membrane and luminal cell accumulation in HGF-stimulated cysts. These data collectively suggest that Chn1 and Chn2 suppress Rac1 at the plasma membrane to maintain the monolayer epithelium. Of note, we could dissect two intracellular events; the luminal cell filling at the apical membrane and extension of a tubular structure at the basal membrane. Since knockdown of Chn1 and Chn2 did not affect extension formation at the basal membrane (Fig. 4), and Rac1 is required for extension (Fig. 1B), searching for molecules or reagents that inhibit the Rac1-dependent extension may lead to a novel treatment for cancer invasion. Live imaging with FRET biosensors will facilitate such cell-based screening.

α1-chimaerin cDNA was originally isolated from brain, and its distribution is restricted to brain and testis [19]. α2-chimearin is also expressed in the brain, and in other organs such as heart, intestine, and lung to a lesser extent [26]. Mice carrying spontaneous mutations in α1 and α2-chimaerin showed a rabbit-like hopping gait, impaired corticospinal axon guidance, and abnormal spinal central pattern generators [27]. β1-chimaerin was originally identified as a testis-specific RacGAP [28]. Using an antibody against β1-chimaerin, the β2-chimaerin isoform was discovered in the brain [29]. β2-chimaerin knockout mice were generated to show that β2-chimaerin is required for presynaptic pruning in the hippocampus [30]. Although the role of Chimaerins in the epithelial structure has not been fully investigated, it has been suggested that β2-chimaerin controls progression of malignant glioma, based on the fact that β2-chimaerin is down-regulated in high-grade glioma [31]. It has also been reported that β2-chimaerin expression was reduced in breast cancer cell lines and tumors [31]. In MDCK cells, both Chn1 and Chn2 are required to maintain the cyst structure (Fig. 3). Although we could not compare protein levels of Chn1 and Chn2, it is speculated that both are equally expressed in MDCK cells, because either Chn1 or Chn2 depletion caused Rac1 activation and luminal cell accumulation. Moreover, it is speculated that the SH2 domain in Chn2 is not required to maintain the cyst structure (Fig. 2E). Since we believe that luminal cell accumulation corresponds to benign hyperplasia in the body, additional mutations or expression changes of other genes will be required for progression. Further clinical surveys of benign and malignant tumors will determine whether Chimaerin can be a target for therapy.

## Supporting Information

Figure S1 **Schematic representation of a rapamycin-inducible Rac1 activity manipulation system (Corresponding to** **Figure 1B** **).** In this system, rapamycin induces the hetero-dimerization of FK506-binding protein (FKBP)-fused proteins and FKBP12-rapamycin binding domain (FRB)-fused proteins. In cells expressing Lyn-FRB, an FRB protein anchored at the plasma membrane, the FKBP-fused GAP domain translocates from the cytosol to the plasma membrane upon rapamycin treatment.
(EPS)

Figure S2 **Schematic representation of a monitor for Diacylglycerol (DAG) (Corresponding to** **Figure 2** **)**. YFP and TFP denote a yellow-emitting mutant of GFP and teal fluorescent protein, respectively. K-Ras4B-CT (blue box) and Gly-Gly indicates the C-terminal region of K-Ras4B and glycine-glycine linker, respectively. The C1 domain of PKCβII was utilized as the DAG-binding domain. In this biosensor design, the FRET level will increase when the biosensor recognizes DAG (right)
(EPS)

Figure S3 **Quantification of Chimaerin Knockdown (Corresponding to** **Figure 3** **).** Total RNA was extracted from MDCK cells expressing shLuc, shChn1, and shChn2. After reverse transcription, relative mRNA levels of Chn1 (A) and Chn2 (B) over control shLuc were quantified. Shown are the averages of three independent experiments with SD (n = 3).
(EPS)

Figure S4 Combined Chn1 and Chn2 knockdown affects cyst morphology (Corresponding to Figure 3C). MDCK cysts expressing both shChn1 and shChn2 together were treated with HGF for 2 days and imaged. Shown are the averages of three independent experiments with SD. The numbers of scored cysts are: Experiment (Exp.) 1 [n = 72 (−HGF), 99 (+HGF)]; Exp2 [n = 61 (−HGF), 87 (+HGF)]; Exp3 [n = 76 (−HGF), 67 (+HGF)]. *P<0.05.
(EPS)

Table S1 **List of GAPs for Rho family GTPases in the microarray analysis.** Genes whose mRNA is upregulated in the late stages (magenta) or downregulated (blue). Genes whose mRNA levels were not certified were labeled with gray.
(EPS)
